# Anchors on prices of consumer goods only hold when decisions are hypothetical

**DOI:** 10.1371/journal.pone.0262130

**Published:** 2022-01-05

**Authors:** Magdalena Brzozowicz, Michał Krawczyk

**Affiliations:** Faculty of Economic Sciences, University of Warsaw, Warsaw, Poland; University of Naples Federico II: Universita degli Studi di Napoli Federico II, ITALY

## Abstract

We elicit willingness to pay for different types of consumption goods, systematically manipulating irrelevant anchors (high vs. low) and incentives to provide true valuations (hypothetical questions vs. Becker-DeGroot-Marschak mechanism). On top of a strong hypothetical bias, we find that anchors only make a substantial, significant difference in the case of hypothetical data, the first experiments to directly document such an interaction. This finding suggests that hypothetical market research methods may deliver lower quality data. Moreover, it contributes to the discussion examining the mechanism underlying the anchoring effect, suggesting it could partly be caused by insufficient conscious effort to drift away from the anchor.

## Introduction

Many of the research methods commonly used to elicit consumers’ willingness to pay (WTP) involve hypothetical questions; in other words, the responses have no financial consequences for the participant. Contingent valuation and conjoint analysis represent two popular examples of such methods. It is well known that hypothetical WTP tends to exceed that of actual WTP, an effect dubbed *hypothetical bias* (HB) in behavioural literature. If the size of the bias was broadly constant across individuals and circumstances, simply calibrating the hypothetical responses would be an easy fix. This, however, is unlikely to be the case. More generally, it is an important, plausible, and rarely investigated proposition that hypothetical data tends to be of lower quality.

In this study, we seek to verify one testable manifestation of the conjecture about the lower quality of hypothetical data; namely, we examine whether the inclination to be driven by useless “hints” (otherwise known as the anchoring effect) is attenuated when the reported WTP has real-life consequences for the participant. Additionally, if this was the case, providing a low anchor could prove to be a simple, hitherto rarely if ever used, way to reduce or eliminate HB in marketing studies.

Of course, our study also contributes to the rich literature on the anchoring effect. Given that it represents a disturbing feature of human judgment and decision-making, it is certainly worth investigating whether it may be lessened when reported values have real consequences. Such an interaction would suggest that the effect may chiefly be understood in terms of insufficient incentives to modify the most directly available value, not in terms of humans’ inherent inability to do just that. The practical implications thereof are substantial. Arguably, affecting (raising) the WTP is *the* goal of marketing and several ways to achieve this are clearly related to the idea of anchoring. Setting an initially high price and then selling at a discount is one common example; calling a small Starbucks coffee *tall*, et cetera, to *disassociate* from the (low) price anchors of competitors is another.

Considering how the interaction between the anchoring effect and the HB affects WTP is thus important for the two areas of literature for both theoretical and practical reasons. Despite this, as we will explore, it has not yet been directly addressed in the empirical literature.

## Literature review

HB can be defined as a systematic difference between valuation amounts observed in declarative research compared to actual WTP [1]. Previous meta-analyses have clearly shown that HB is ubiquitous and substantial. Summarising 77 studies, Foster and Burrows [2] identified that the median hypothetical WTP values exceed those observed in real trials by as much as 39%. Of course, it would be an oversimplification to consider the value reported in incentivised trials as “real”. However, some studies have at least shown that they are better predictors of actual market behaviour [3].

As can be expected, the HB is to some extent moderated by the specific choice of research method. The literature shows substantial heterogeneity in this respect. Popular methods for obtaining the hypothetical value include direct open-ended questions [4], choice experiments [5], and declarative Vickrey auctions [6]. Likewise, real values have been, among other ways, elicited using *n*-th price auctions [7], the Becker-DeGroot-Marschak (BDM) procedure [8], and the implementation of a randomly chosen decision card in the context of discrete choice modelling. Several investigations have refrained from eliciting specific WTPs altogether, instead only observing purchase/abstain decisions instead [9]. The general finding is that choice-based elicitation methods tend to reduce bias [10, 11]. Some researchers have focused on the effect of the sample (using students may contribute to the bias, [10]) and the goods in question (finding HB to be weaker in private goods compared to public goods, [12]).

HB may arise due to many various factors—there is no one widely accepted theory. One of the reasons why people behave (and report WTP value) differently under hypothetical conditions may be the fact that participants are aware that their behaviour is being scrutinized and they may seek to provide answers that are socially acceptable [13]. Of course, this tendency may be observed also in incentivized conditions. However, the incentives to report the truth may counterbalance the effect, thus the problem is expected to be more severe in the hypothetical conditions. Another factor that might distort hypothetically reported values, especially in dichotomous choice or referendum format surveys, is the tendency to avoid the cognitive dissonance that occurs when the respondent’s WTP is positive but lower than the bid amount. In this case, participants worry that their truthful answer “No” may signal the interviewer that they do not value the good or service at all. This may compel them to vote "Yes" even if the bid is higher than their actual WTP, a case of HB [14]. Some researchers suppose that respondents overstate their WTP, because they gain utility from pleasing the experimenter or interviewer or from creating a positive self-image [15]. The studied bias may be also explained by the intention-behaviour discrepancy [16].

In some contexts, notably in the case of public goods and services, hypothetical questions may be the only option. Thus, a number of studies have addressed the question of mitigating HB. Two types of approaches used to minimize it can be distinguished, namely *ex ante* and *ex post* techniques. In the first group, there are several approaches for reducing HB. One of them is a technique called *cheap talk* [4, 6] in which the respondents are explicitly informed about the existence of HB in previous surveys and instructed not to overstate their WTP. Another approach involves making the design of a survey consequential [17]. Next, in the Honesty and Realism approach [18] participants are asked to provide the true and honest answers (or they even sign a special oath). As the reason for overestimation of WTP values may be mentioned before *social desirability bias* and *cognitive dissonance*, scholars have also developed procedures designed for reducing those phenomena, such as “inferred valuation” (respondents are asked what they think others would pay for the good) or trichotomous choice (participants are allowed to vote in favour of a project at an amount less than their original bid [18, 19]). The ex-post approaches to reduce HB include various calibration techniques, eg. data screening, related market calibration, uncertainty recoding [20].

Another strand of literature which is relevant to the current research concerns anchoring (see [21] for a review). This effect is obtained when a seemingly irrelevant number (an anchor) is shown to the participant and affects his or her numeric judgment—forecast, estimation, or evaluation. The effect was first explored by Tversky and Kahneman [22]); in their laboratory experiments, participants were asked whether the fraction of African countries belonging to the United Nations was higher or lower than the outcome on a wheel of fortune they observed, before providing their best guess. This final answer was strongly correlated with the (obviously irrelevant, exogenously random) outcome on the wheel of fortune.

Tversky and Kahneman proposed that the participants anchor on the signal and adjust upwards if it is deemed too low and downwards if it is deemed too high. However, this adjustment tends to be insufficient, because it is stopped when the plausible range is reached. That is, a participant may have an idea that the fraction is probably somewhere between 40% and 70%. If an anchor of 30% is provided, the participant says that the target value is higher than the anchor and subsequently reports that it is approximately 40%. For an anchor of 80%, the participant indicates that the value is lower than that, perhaps somewhere close to 70%.

A prominent alternative explanation (although the two mechanisms are not mutually exclusive) was provided by Strack and Mussweiler [23]. These authors claim that anchors affect subsequent information retrieval: anchor-consistent information is more easily accessible, so that the ultimate response tends to be close to the anchor. The subsequent literature provided further theoretical insights that are beyond the scope of this paper. One important but contested proposition is that adjustment from the anchor requires conscious cognitive effort. Among its testable predictions, it has been reported that cognitive load amplifies the anchoring effect [24, 25].

Diverse empirical studies have been orchestrated, with anchoring being observed for estimates of temperatures in Antarctica [26], the probability of a nuclear war [27], judgment tasks [28], estimates of project duration [29], lottery evaluation [30], and price estimates (e.g., [31]). From the point of view of this study, the most important are the results of studies concerning declared preferences, especially WTP for specific goods. This strand of the literature is also vital from the economic and marketing viewpoints, as anchoring affects not only the average but also the whole distribution of WTP values and, consequently, the demand curve [32]. Early examples include Johnson and Schkade’s [33] experiment involving certainty equivalents of abstract lotteries and studies by both Green et al. [34] and Kahneman and Knetsch (The Effect of Format. Unpublished manuscript. University of California, Berkeley. 1993) for WTP for public goods.

In an influential paper, Ariely et al. [35] identified very strong anchoring effects on the valuation of unpleasant hedonic experiences (annoying sounds), as well as some common consumer goods, such as small electronic items and wine. However, subsequent work suggested the limited robustness of these results. Bergman et al. [36], Tufano [37], and Maniadis et al. [38], discovered weaker effects, whilst Simonson and Drolet [39] only observed the moderate impact of anchors on WTP (but not Willingness to Accept, WTA) for consumer goods; Sugden et al. [40] found that only plausible (not extreme) price anchors affect WTA (and, to a weaker extent, WTP); Fudenberg et al. [41] reported minor effects for consumer goods but none for random lotteries; Alevy et al. [42] found no effect on WTP for peanuts and collectible sports cards.

Then, while the anchoring effect is regarded to be robust in numeric judgment tasks (e.g., concerning general knowledge), its effect in valuation tasks was shown to be inconsistent. Yoon et al. [43] examined the robustness of anchoring effects on preferences (measured by WTP). Based on an analysis of earlier studies and on their own experiments, they found that differences between experimental procedures cannot explain the inconsistencies in the existing literature, and that the anchoring effect is generally robust to experimental settings. Their findings were only partially confirmed in a recent meta-analysis of anchoring studies on WTA or WTP: Li et al. [44], based on 53 studies from 24 articles, obtained an effect of moderate size. Moreover, they concluded that the strength and robustness of the phenomenon might not be as great as previously believed. Ioannidis et al. [45], in the recent paper questioning the robustness of the anchoring effect on preferences, observed no effect of anchors on the valuation of a bottle of wine. They also performed a concise meta-analysis and demonstrated that the anchoring effect is weaker for familiar goods than for unfamiliar ones and also observed a null result of anchoring in studies with clearly uninformative anchors.

Some anchoring studies (e.g., [29]) have used (non-trivial) real incentives, but none investigated how incentives to provide the “correct” value change the working of anchors by direct experimental manipulation, keeping other factors constant. That, of course, would be most relevant for our purpose. In the body of literature examining anchors and incentives, it is often claimed that incentives are not important, but in fact the evidence is scarce and often reported merely in passing. For example, Tversky and Kahneman [22] devote exactly one sentence to the issue: “Payoff for accuracy did not reduce the anchoring effect”. We are thus not told the number of participants, which questions were incentivized, nor the nature of incentives, among other matters. Chapman and Johnson [46] are equally enigmatic: “Chapman and Johnson ([still] unpublished data) used a procedure in which half the subjects were paid according to the preferences implied by their judgments of simple lotteries. There was no reduction in anchoring when subjects’ payoffs depended upon their responses”. Likewise, Wilson et al. [47] only sketched the design of Study 4, in which participants judged the number of surgeons and physicians in the phone book and were rewarded for accuracy.

The experimental procedures of Epley and Gilovich [48] are reported more comprehensively. They investigated two types of anchors: standard ones, i.e., explicitly given by the experimenter, and self-generated ones (e.g., the year 1492 was a natural self-generated anchor for the question about the year in which the *second* European explorer landed in the West Indies provided by the experimenter). They argued (although [49] disagreed) that incentives (which were modest and manipulated between-subjects) could act differently depending on these two variations of the anchoring effect. Indeed, they observed that only self-generated anchors were weakened by monetary rewards. In a very recent study, Enke et al. [50] tested the effect of incentives on four well-known cognitive biases, including anchoring effect. In none of the tasks did they observe the incentives to reduce the bias.

It should be emphasised that all of the aforementioned studies on anchors and incentives involved judgment rather than valuation tasks. Moreover, these judgment tasks could typically be considered to be somewhat interesting or entertaining.

One plausible reason why (modest) real incentives might have made little difference was that responders were intrinsically motivated to guess correctly even in hypothetical cases. Indeed, one typically wants to answer correctly provided there is a well-defined “right” and “wrong”. The WTP questions are different in this respect, because here, conversely, the response is largely a matter of personal preference. Incentives may thus make a difference in these kinds of questions, even if they do not in the case of standard judgment tasks.

In the aforementioned meta-analysis of studies measuring WTP and WTA, Li et al. [44] reported that incentives do not attenuate the anchoring effect. However, a large majority of the included studies were hypothetical; the few that used incentives could also differ on other dimensions, reducing the statistical power to detect an effect.

The only other project we are aware of that provides a direct comparison of *valuations* of analogous objects under hypothetical and real scenarios is Jung et al. [51]. In a series of field experiments, the authors investigated the impact of anchors on the amounts submitted by customers under the pay-what-you-want (PWYW) system, some of which were replicated as hypothetical scenarios in the laboratory (studies 14a-d). They suggest that some moderators of anchoring may operate in the hypothetical scenario only and that hypothetical payments are more sensitive to anchors than real ones. However, it must be emphasised that customers *cannot* be expected to offer their true WTP in a PWYW system. In this sense, while there were real incentives, there was no incentive compatibility, i.e., it was generally not in any participant’s best interest to reveal their own preference by offering an amount corresponding to their actual WTP [16]. Specifically, offering little in a PWYW system could be a signal not only of not being interested in the product, but also of being selfish. It is perhaps not very surprising that when the participants get a hint that it is appropriate to offer a lot (a high anchor), they are willing to comply to avoid sending a signal that they are selfish, but more so when this can be done at no cost (as in the hypothetical condition).

We attempted to address this research gap in our project. In the first attempt (working paper; blinded for review) we elicited WTP for a mascara in two laboratory experiments involving both hypothetical valuation and second-price sealed-bid (Vickrey) auctions with real transactions. However, we observed no anchoring effect even in the hypothetical condition. Therefore, we were not in a position to investigate if the anchoring effect becomes smaller, in relative terms, when real incentives are provided. Looking at the result thus, one cannot tell what (if any) reduction took place. Therefore we do not include these data in the current report, instead focusing on the studies in which, thanks to important changes in the methods and stimuli, we were able to trigger a reliable anchoring effect in hypothetical choices.

## Research hypotheses

We sought to verify the following main hypotheses:

**H**_**1**_ **: HB influences the valuation of the good**

Based on the extant literature, we expected that the participants we assigned to make a purely declarative decision would report a higher WTP for the good than those making actual purchasing decisions.

**H**_**2**_ **: The anchoring effect influences the valuation of the good**

We expected that participants who were shown a high anchor would report a higher WTP for the good than those shown a low anchor.

**H**_**3**_ **: HB and the anchoring effect interact**

We expected that anchoring would be stronger in the hypothetical case than in the case of actual purchasing decisions.

In view of the insufficient adjustment explanation of the anchoring effects, adjustment from the anchor is a process that requires cognitive effort [52]. Financial incentives to make this effort should therefore attenuate the anchoring effect.

Our expectations are also in line with a heuristic-systematic model of persuasion [53]. The basis of this theory is the distinction between systematic and heuristic information processing. Systematic processing is defined as an extensive, analytic approach in which all informational input is accessed and integrated. This type of processing is assumed to demand an adequate level of effort and cognitive capacity. According to this model, therefore, when people are not sufficiently motivated to make the cognitive effort, they use heuristics or other hints to determine their judgments, because heuristic processing requires much less cognitive effort than systematic processing.

## Design and procedures: The elements common to all the experiments

To test these hypotheses, three experiments were conducted. In each of them, the participants were asked to state their WTP for a presented product.

In each experiment, four different treatments were used in a 2x2 design: BDMLow (real transactions, low anchor), BDMHi (real transactions, high anchor), HypoLow (hypothetical valuation, low anchor), and HypoHi (hypothetical valuation, high anchor).

Under hypothetical conditions, the technique of directly eliciting the WTP value was used (a simple request: “give the maximum price at which you would be willing to buy the presented product”), with the participants understanding that their valuation was declarative only. By contrast, the incentive-compatible BDM technique [54] was used in the BDM conditions (similarly as in [55, 56]). The BDM procedure, being fairly simple and transparent, easy to orchestrate and relatively cheap, is commonly used in laboratory experiments, but also enables experimenters to elicit consumers’ WTP in typical purchase settings in the field [55]. Almost all participants declared that the procedure was understandable and clear. Moreover, BDM procedure seems to be very well-suited to our field experiments. Because we were dealing with just one subject at a time, we could not have used second-price auctions; we also wanted to avoid long response forms that would have been necessary for a multiple price list approach. Therefore, as we wanted to use the same approach in all experiments, we selected BDM procedure for both laboratory and field experiments. In this procedure, we asked participants to name the maximum price they would be willing to pay for the presented product, before drawing the transaction price from a pre-specified distribution (in the form of small cards placed in a box). The range of the distribution from which the exogenous price was drawn was not revealed to the participants to avoid alternative anchoring [57]. If the participant’s offer was higher than or equal to the drawn price, the participant was required to buy the product at the drawn price. If the offer was lower than the drawn price, the transaction was not executed. The weakly dominant strategy in this procedure is to state the true WTP [55].

Because all the experiments involved adult volunteers, who were not deceived or exposed to a real risk of psychological or physical harm, the approval of the Committee of Research Ethics, Faculty of Economic Sciences, University of Warsaw was waived.

## Experiment 1

Experiment 1 was a paper-and-pencil laboratory experiment in which participants were asked to state their WTP for a voucher for a black-and-white caricature or portrait in A4 format, painted by an experienced artist. They could acquire this voucher for themselves or for somebody else. It was valid for three months. We predicted that most participants would not have frequently purchased such goods. They would thus find it difficult to judge how much they would like the product or how much it would normally cost. Similarly, we expected this type of product would be sufficiently susceptible to anchoring.

The assignment to a BDM vs. Hypo condition was randomised at the session level for the sake of convenience and to exclude the possibility of treatment contamination, whereas a Hi or Low anchor was randomly assigned to each individual within a session. At the beginning of the session, participants were informed about the basic principles of the experiment (see S1 Appendix for instructions) both orally and in writing. The voucher was then presented to them and some examples of the artist’s works were shown. Participants were asked whether they would pay 20 PLN (Low anchor) or whether they would pay 80 PLN (High anchor) for the voucher. These values were informed by the range of prevailing market prices charged by unknown artists (and analogous statements are true for the specific values used in the other two experiments). In the BDM condition, the participants then took part in a BDM procedure. To avoid long lines, the BDM procedure was actually only implemented for three randomly chosen participants in each session (about 10–20% of participants). For each of these three participants, the reported WTP was compared to the randomly selected transaction price. If they were equal or the WTP was higher, the participant in question obtained the voucher, paying for it with their own money. All participants were aware this would be the case, but did not know who would be randomly chosen, thus had to make their decisions under the assumption that they may be consequential. At the end of the experiment, the participants were asked to fill in the post-experimental questionnaire covering, among other things, their sociodemographic characteristics and interest in art; see S2 Appendix for the translation. In the Hypothetical condition, the scheme was analogous, but the BDM procedure was not used; the participants simply provided their valuations in response to an open-ended hypothetical question.

The experiment was conducted at the Faculty of Economics of the University of Warsaw. Because participants were recruited immediately after their classes and the sessions took approximately 15 minutes, no show-up fee was deemed necessary. In total, 218 local students took part, 53% of which were female. The mean age was 19.5 years; a typical participant was a single person with no employment, in a good financial situation.

In Table 1, we compared socio-demographic characteristics of respondents in each treatment.

**Table 1:** Experiment 1: Comparison of respondents’ characteristics across all treatments.

		HypoLow	HypoHi	BDMLow	BDMHi	*P*
Gender	Female	54.90%	57.69%	46.55%	52.63%	0.7733
	Male	45.10%	42.31%	53.45%	47.37%	
Age	Mean	19.39	19.62	19.55	19.56	0.8263
	Median	19	19	19	19	
Place of birth	Village	13.73%	17.31%	20.69%	19.30%	0.9321
	City	86.27%	82.69%	79.31%	80.70%	
Employment	Employed	19.61%	21.15%	22.41%	28.07%	0.8799
Relationship	Single	64.71%	65.38%	70.69%	63.16%	0.9079
	In a relationship	35.29%	34.62%	29.31%	36.84%	
Financial situation	Very bad, bad or moderate	45.10%	42.31%	43.10%	40.35%	0.9797
	Good or very good	54.90%	57.69%	56.90%	59.65%	

As can be seen in Table 1, the distribution of the respondents’ characteristics differed very little across treatments. We verified our observations using the Kruskal-Wallis equality-of-populations rank test. All tests showed the equality of populations in all treatments (with p>.7), indicating a successful random assignment.

### Results of Experiment 1

**Fig 1:**
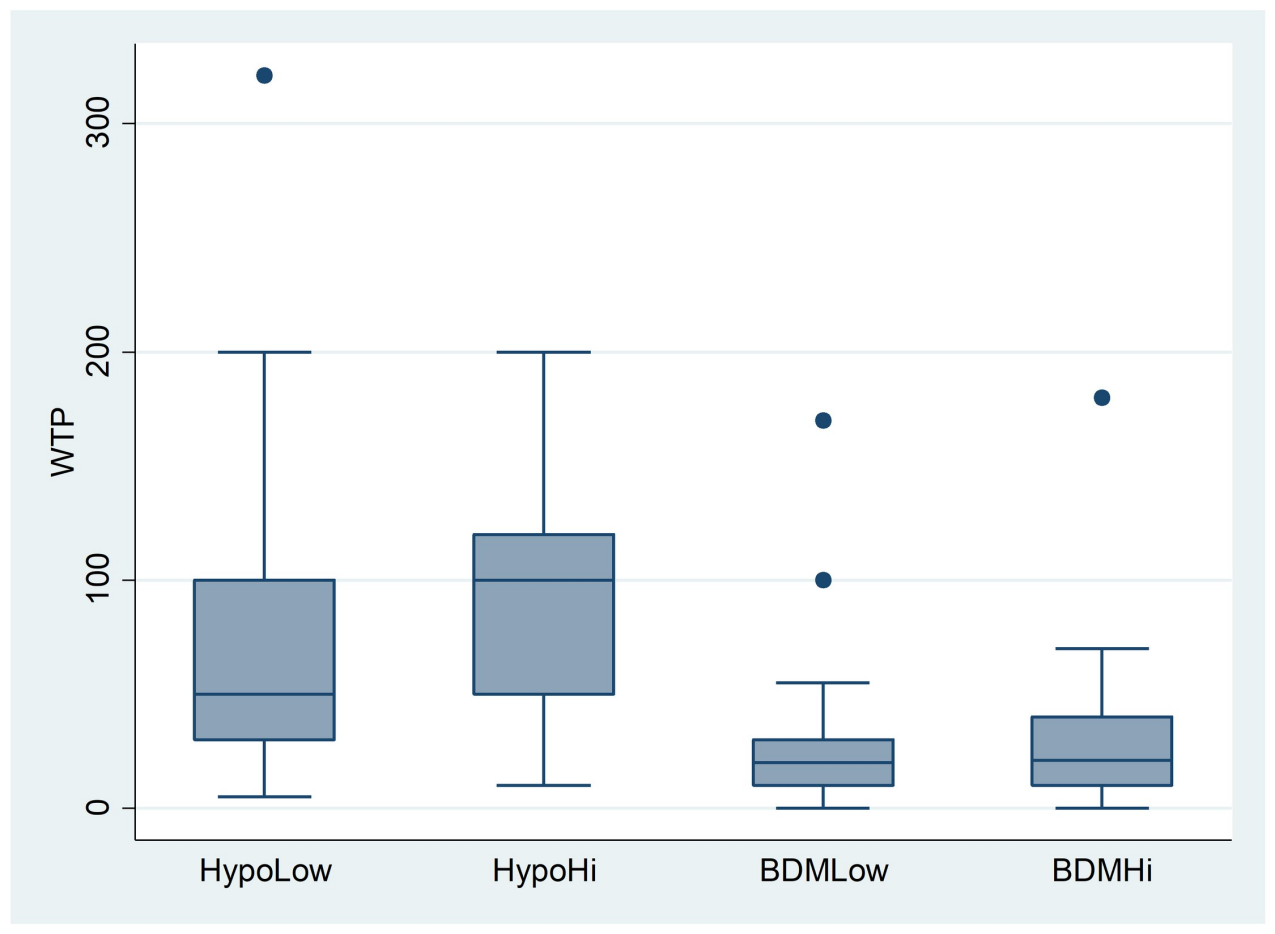
Experiment 1: WTPs by treatment (in PLN). The blue rectangle represents the middle 50% of the data (from the first quartile to the third), the line inside the box shows the median (the second quartile). The whiskers represent the top and bottom 25% values, excluding outliers, which are represented by dots.

The mean value of WTP for the voucher in the whole sample was equal to 58.32 PLN (12.88 EUR), with a median value of 40 PLN and a standard deviation of 65.83 PLN (for histogram, see S1 Fig). These aggregate measures mask substantial differences between the treatments, however. We started our investigation by comparing WTP values in each treatment, see Table 2 and Fig 1.

**Table 2:** Experiment 1: WTPs by treatment (in PLN).

	HypoLow	HypoHi	BDMLow	BDMlHi
**Mean**	82.08	98.10	30.04	29.03
**Median**	50	100	20	21
**Standard dev.**	86.83	56.94	50.46	28
**N**	51	52	58	56

From a visual inspection alone, the mean WTP was lower in the Real condition than in the Hypothetical condition. The anchor made a difference in the Hypothetical treatments, but not in the BDM treatments. We verified these observations using non-parametric Mann-Whitney U tests. First, we compared treatments with a Hypothetical vs. BDM valuation to test for HB (separately for the low and high anchors). The difference between the WTP values in the HypoLow vs. BDMLow treatments was statistically significant (z = 5.486, p< .0001), and likewise for the HypoHi vs. BDMHi treatments (z = 7.075, p< .0001). Thus, we observed a clear HB. In the next step, we compared treatments with low and high anchors (separately for hypothetical and auction valuations) to verify the anchoring effect. The difference between the WTP values in the HypoLow and HypoHi treatments was statistically significant (z = −2.737, p = 0.0062). By contrast, there was no difference in the case of real valuations (z = −1.145, p = 0.2520). In light of these results, we can state that the anchor affected the hypothetical valuation of vouchers (raising the mean WTP value by approximately 20%) but had no impact on valuations in the case of real transactions.

To explicitly test for the interaction, we conducted a regression analysis. We estimated the Ordinary Least Squares regression models with the WTP value as the dependent variable (see Table 3 for variable labels).

**Table 3:** Experiment 1: Variable labels.

hypothetical_dummy	1—for declarative (hypothetical) valuation,
	0—for real valuation
high_anchor_dummy	1—for a high anchor,
	0—for a low anchor
hypothetical#high_anchor_interaction	interaction between hypothetical and high_anchor dummies
male	1- male,
	0—female
in_relationship	1—if the participant is in a relationship,
	0—if the participant is single
unemployed	1- if the participant is unemployed,
	0—otherwise
city	1- if the participant’s place of birth is a city,
	0—if the participant’s place of birth is a village
financial_situation	1- if the participant is in a good or very good financial situation,
	0—in all other cases
likes_examples	1- if the participant likes or really likes the examples of the artist’s work,
	0—otherwise
gift	1—if the participant would like to use the voucher as a gift,
	0—in all other cases
art	1- if the participant is interested in art,
	0—otherwise
price_caricature	the perceived market price of a caricature
price_portrait	the perceived market price of a portrait

The dependent variable was logarithmised (in order to obtain the correct functional form of the presented models). As in model 5 we detected the heteroscedasticity problem, we used the White’s robust covariance matrix there (in models 1–4 the assumption that residuals are homoscedastic was met). All of the models included the experimental conditions (*hypothetical*_*dummy* and *high_anchor_dummy)*. In model (2), we also included the interaction between the two. In model (3), we additionally controlled for certain sociodemographic characteristics such as gender, age, or place of origin. Model (4) further included participants’ self-reported interest in art, the extent to which the participant liked the example work shown to them previously and whether (s)he intended to offer the voucher for the portrait/caricature as a gift, should (s)he buy one. Finally, in model (5), we also included perceived market price of the caricatures and portraits. Table 4 presents all regression models.

**Table 4:** Experiment 1: Regression table: WTP values.

	(1)	(2)	(3)	(4)	(5)
**hypothetical_dummy**	1.222***	1.149***	1.145***	1.086***	1.090***
**high_anchor_dummy**	0.351***	0.278	0.279	0.325	0.380
**hypothetical#high_anchor_interaction**		0.149	0.167	0.142	0.082
**male**			0.113	0.174	0.179
**age**			-0.108	-0.099	-0.101
**in_relationship**			0.250	0.218	0.137
**unemployed**			0.107	0.130	0.075
**city**			-0.170	-0.116	-0.133
**financial_situation**			0.182	0.153	0.165
**likes_examples**				0.439***	0.405***
**gift**				-0.009	0.008
**art**				0.182	0.121
**price_caricature**					0.004
**price_portrait**					0.000
**cons**	2.786***	2.821***	4.749***	4.173***	4.026***
**N**	211	211	211	211	205
**R-sqr**	0.3164	0.3174	0.3491	0.3917	0.4335
**F**	48.13	32.09	11.98	10.63	14.96
**Prob>F**	0.0000	0.0000	0.0000	0.0000	0.0000

** p < .05

*** p < .01

OLS models did not confirm that the interaction between our experimental dimensions, *hypothetical#high_anchor_interaction*, was statistically significant. However, we can also notice that the pure effect of anchor in the BDM condition is not statistically significant either. The remaining variables in our models affected the WTP in natural ways; in particular, it was higher in those who liked the artist’s works on display. The Wald post-estimation test (see S3 Appendix) also indicated that we cannot reject the hypothesis of the lack of interaction (F(1, 207) = 0.32; p = 0.5698).

However, the same OLS models estimated in the sub-samples based on the method of WTP elicitation—hypothetical vs. BDM proved that the effect of the high anchor was statistically significant in the hypothetical sub-sample, but was not significant in the BDM sub-sample, which is in line with the results of Mann-Whitney tests presented before; for models see S4 Appendix.

Because the data is not normally distributed, we applied yet another test of the interaction between the anchoring effect and the HB, namely the permutation test for analysis of variance (permutation ANOVA). We can notice that neither the interaction between the two variables of interest nor the pure effect of anchoring in the BDM treatment were statistically significant; Table 5 shows the results.

**Table 5:** Experiment 1: Permutation ANOVA.

Source	SS	df	F	parametric P>F	permutation p> F
hypothetical	198373	1	87.8373	<0.0001	0.0002
high_anchor	3045	1	0.8878	<0.0001	0.3550
hypothetical#high_anchor	3923	1	1.1438	<0.0001	0.2854
Residuals	730557	213			

To summarise, a large HB was found. Further, the anchoring effect was observed in the hypothetical answers, but not when real money was at stake. However, the OLS models and ANOVA did not directly evidence the statistically significant effect of interaction between incentives and anchor. The results thus seem to be somewhat ambiguous; the explanation of which may be the outliers that are found in our data and can distort the analysis. Moreover, the sample size of Experiment 1 was relatively small and comprised only students. To verify robustness of the interaction effect, we therefore conducted two further studies.

## Experiment 2

This study was a field experiment conducted in one of the largest shopping centres in Central Europe, Westfield Arkadia in Warsaw in September 2020. The participants were asked to state their WTP for a ceramic mug painted by an experienced artist. This product is potentially useful and desirable for customers of all ages and genders. Moreover, such mugs widely vary in price depending on the painting technique used, design, author’s artistic talent, etc. We could thus expect it to be susceptible to anchoring, as participants would not know the actual market price of the valued good.

Before the experiment, we carried out an online survey in which the participants (*N* = 52, diversified in terms of age and sex) were asked to pick the three most attractive designs of the 11 options presented. For the main experiment, we selected the set of three mugs (see S2 Fig) that maximized the fraction of the pilot study participants that picked at least one of the three. This fraction was as high as 80%. Moreover, all but one of these participants rated the mugs eventually used in the main experiment as a “3” or “4” on a 1–4 scale.

The selected mugs were presented at a professionally prepared stand, located in a part of the shopping center with a very high foot traffic. The stand operated during the opening hours of the shopping center. The customers passing by, encouraged by the display of presented products, the banner promising a chance to win a prize (a 100 PLN Westfield Arkadia gift card), and sometimes a queue, approached the stand and were randomly assigned to individual experimental conditions.

In each treatment, at the beginning the participants were orally informed about the basic principles of the experiment (see S5 Appendix for instructions). We then showed them the mugs, enclosing additional information about the product and the artist. The participants were asked to choose which of the three mugs that they liked the most. They were then asked whether they would pay 10 PLN (in the Low anchor condition) or whether they would pay 60 PLN (in the High anchor condition) for the selected mug. Then, the participants were asked to state their WTP. Under hypothetical conditions, the technique of directly eliciting the WTP value was used, the participants were informed that their valuation was declarative only. By contrast, the incentive-compatible BDM technique (Becker et al., 1964) was used in the BDM condition.

For our participants’ convenience, we accepted both payment in cash and by card. At the end of the experiment, the participants in each treatment were asked to complete a short post-experimental questionnaire providing additional information on their socio-demographic characteristics (see S6 Appendix for the questionnaire). After that, the participants performed a task involving recreating on a touch screen a shape they briefly saw. First, each participant selected one shape from the three shown on the screen and looked at it for 10 seconds. The shape then disappeared and the participant had 30 seconds to redraw it from memory. Sufficiently precise drawings (evaluated by the application) were rewarded with a 100 PLN gift card. This way of allocating the participation fee turned out to be appealing and entertaining for the participants.

A typical experiment took approximately 10 minutes. In total, 786 shopping center customers (aged 16 and over) took part. The research sample was naturally diversified in terms of age, sex, education, etc. Roughly 53% of participants were female, with a mean age of 32 years. Similar to Experiment 1, we compared the basic sociodemographic characteristics of the respondents assigned to different treatments, see Table 6.

**Table 6:** Experiment 2: Comparison of characteristics of the respondents in all treatments.

		HypoLow	HypoHi	BDMLow	BDMHi	*p*
Gender	Female	48%	58.88%	53.54%	53.40%	0.3178
	Male	52%	41.12%	46.46%	46.60%	
Age	Mean	31.29	31.16	32.48	33.17	0.4122
	Median	27	26	26	27	
Education	Primary	12%	10.66%	5.56%	6.81%	0.7865
	Vocational	4%	3.05%	3.54%	3.14%	
	Secondary	31%	35.03%	39.90%	34.03%	
	Higher	53%	51.27%	51.03%	56.02%	
Employment	Employed	72.50%	72.59%	71.72%	70.68%	0.9871

We can see that the distribution of particular answers is similar across all treatments. Again, we carried out Kruskal-Wallis equality-of-populations rank tests. The tests indicated the same distribution of all measured characteristics across all treatments.

### Results of Experiment 2

**Fig 2:**
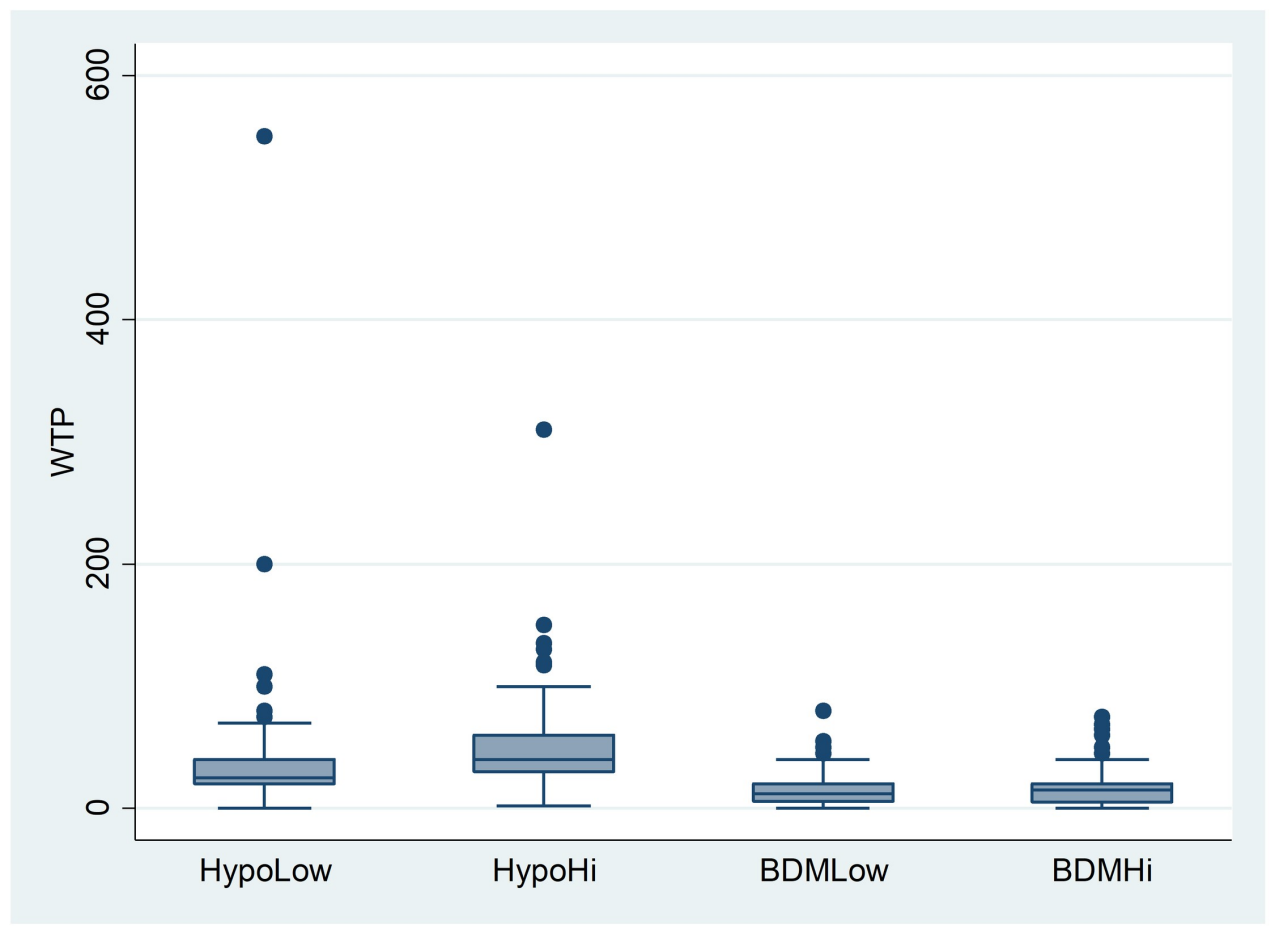
Experiment 2: WTPs by treatment (in PLN).

The mean WTP value for the ceramic mug across the sample was equal to 29.11 PLN (6.34 EUR), with a median value of 20 PLN and a standard deviation of 32.24 PLN (for histogram, see S3 Fig). Again, we first compared the WTP values in each treatment; see Table 7 and Fig 2.

**Table 7:** Experiment 2: WTPs by treatment (in PLN).

	HypoLow	HypoHi	BDMLow	BDMHi
Mean	33.97	49.39	15.48	16.19
Median	25	40	12	15
Std. Dev.	42.99	33.96	12.54	13.55
N	200	197	198	191

Again, from a visual inspection alone, the mean WTP is higher in the Hypo condition than in the BDM one. The anchor affects WTP values in treatments with hypothetical valuations, but not in treatments with real transactions. As in Experiment 1, we verified these observations using non-parametric Mann-Whitney U tests. Again, we compared treatments with a Hypothetical vs. BDM valuation to test for HB. The differences between the WTP values in the HypoLow vs. BDMLow treatments were statistically significant (*z* = 9.947, *p*<.0001), and likewise in the HypoHi vs. BDMHi treatments (*z* = 13.219, *p*<.0001). Thus, we can state that HB affects the valuation of the presented mugs. In the next step, we compared treatments with low and high anchors to verify the anchoring effect. The differences between the WTP values in the HypoLow and HypoHi treatments were statistically significant (*z* = −6.504, *p* = <0.0001). By contrast, there was no difference in the case of real valuations (*z* = −0.284, *p* = 0.7765). The anchor affected the hypothetical valuation of vouchers (raising the average WTP values by about 53%) but had no impact on valuations in the case of real transactions.

We once more verified these findings using OLS regression models. Table 8 provides variable labels.

**Table 8:** Experiment 2: Variable labels.

hypothetical_dummy	1—for declarative (hypothetical) valuation,
	0—for real valuation
high_anchor_dummy	1—for a high anchor,
	0—for a low anchor
hypothetical#high_anchor_interaction	interaction between hypothetical and high_anchor_dummies
male	1- male,
	0—female
age	participant’s age
higher_secondary_education	1—if the participant has higher or secondary education,
	0—if the participant has primary or vocational education
unemployed	1- if the participant is unemployed,
	0 –otherwise
gift	1—if the participant would like to use the mug/honey as a gift,
	0—in all other cases
mugs_very_nice	1- if the participant assesses the presented mugs as very nice,
	0—in all other cases
experimenter	1—if the experiment was conducted by experimenter no. 1,
	0—if the experiment was conducted by experimenter no. 2
mug_elephant	1- if participant selected the mug with an elephant;
	0 –otherwise
mug_cat	1- if participant selected the mug with a cat,
	0 –otherwise

Again, we used the logarithmized WTP as a dependent variable; the RESET test evidenced that the functional form of all presented models was correct. Because of the heteroskedasticity detected in our data, we used a White’s heteroskedasticity consistent covariance matrix estimators. Models 1–3 were analogous to the specifications in Experiment 1. Model (4) additionally included the extent to which the participants liked the presented mugs, as well as whether participants intended to offer the mug as a gift. Finally, in model (5), we also included the experimenter who carried out the particular experiment and which mug was selected by participants. Regression models are presented in Table 9.

**Table 9:** Experiment 2: Regression table: WTP values.

	(1)	(2)	(3)	(4)	(5)
**hypothetical_dummy**	1.052***	0.835***	0.811***	0.770***	0.778***
**high_anchor_dummy**	0.214***	-0.011	-0.010	0.001	0.005
**hypothetical#high_anchor_interaction**		0.440***	0.458***	0.457***	0.452***
**male**			-0.103	-0.090	-0.087
**age**			-0.010***	-0.011***	-0.011***
**higher_secondary_education**			-0.207	-0.181	-0.183
**unemployed**			-0.170**	-0.170**	-0.166**
**gift**				0.073	0.070
**mugs_very_nice**				0.284***	0.279***
**experimenter**					-0.026
**mug_elephant**					0.043
**mug_cat**					-0.009
**cons**	2.337***	2.448***	3.059***	2.885***	2.896***
**N**	775	775	769	769	769
**R-sqr**	0.2926	0.3048	0.3480	0.3695	0.3701
**F**	168.80	123.69	74.19	62.31	47.54
**Prob>F**	0.0000	0.0000	0.0000	0.0000	0.0000

** p < .05

*** p < .01

In all the models allowing for the interaction (*hypothetical#high_anchor_interaction*), it was highly significant, while the main anchoring effect was not. The results were confirmed by the post-estimation Wald tests (F(1, 771) = 13.61; p = 0.0002), for details see S7 Appendix. We also identified that older participants reported lower WTP values than younger ones and employed participants reported higher WTPs than unemployed ones. Unsurprisingly, WTP was also higher in participants who very much liked the presented mugs.

All the presented results were robust to the exclusion of inconsistent responses, i.e., those in which the reported WTP was lower than the anchor price that was accepted or higher than the anchor price that was rejected. This is not surprising given that only 2.29% of all responses showed this characteristic. While inconsistencies were more common in the Hypothetical condition than in the BDM one, the difference was not statistically significant.

In the last step, as in the previous experiment, we applied the permutation ANOVA. Again, the interaction between the anchoring effect and HB was statistically significant, while the main effect of anchor (in the BDM condition) was not; see Table 10 for the results.

**Table 10:** Experiment 2: Permutation ANOVA.

	SS	Df	F	Parametric P(>F)	Permutation P(>F)
Hypothetical	37801.00	1	44.8158	0.0000	0.0002
high_anchor	47.89	1	0.0568	0.0000	0.8198
hypothetical*high_anchor	9231.42	1	10.9445	0.0000	0.0004
Residuals	659597.55	782			

## Experiment 3

Experiment 3 was also conducted in Westfield Arkadia shopping center in September 2020, but it did not overlap with Experiment 2. As the experiments were anonymous, we cannot verify how many people participated in both but given that there were few cases of perfectly matching demographic characteristics, we conclude that the overlap of the samples was very small indeed.

The design of Experiment 3 was very similar to that of Experiment 2; the key difference was that, instead of hand-painted mugs, we offered jars of flavored honey. After analyzing the popularity of various types, we selected three types: raspberry, garlic & ginger, and cocoa (see S4 Fig). These types of honey are rarely accessible in shops, so we expected that most customers would be unaware of the market price of the offered product.

We used the same stand and location as in Experiment 2. We implemented the same 2x2 design as before to elicit the WTP for the type of honey they were most interested in. Before the participants were asked to state their WTP, they were again questioned whether they would pay 10 PLN (Low anchor) or whether they would pay 60 PLN (High anchor) for the selected honey. Similarly as in Experiment 2, customers were encouraged to take part in the experiment by the banner promising a chance to win a prize (again a 100 PLN Westfield Arkadia gift card) and display of the presented products.

The experiment took approximately 10 minutes per participant. In total, 799 shopping center customers took part; 54% of participants were female and the mean age was 31 years.

As in previous experiments, we start our analysis by comparing respondents’ characteristics across all treatments, see Table 11.

**Table 11:** Experiment 3: Comparison of characteristics of respondents in all treatments.

		HypoLow	HypoHi	BDMlLow	BDMHi	*p*
Gender	Female	58.05%	46.34%	52.04%	59.07%	0.0974
	Male	41.95%	53.66%	47.96%	40.93%	
Age	Mean	31.93	32.04	31.85	29.77	0.0531
	Median	27	27	27	24	
Education	Primary	8.29%	10.24%	9.69%	12.44%	0.3830
	Vocational	2.93%	1.95%	5.10%	1.55%	
	Secondary	35.61%	33.17%	32.65%	37.31%	
	Higher	53.17%	54.63%	52.55%	48.70%	
Employment	Employed	72.68%	69.61%	72.45%	65.28%	0.5542

We can notice that the fraction of females was slightly lower in HypoHi treatment than in the other treatments, and the participants were slightly younger in BDMHi treatment than in the other treatments. However, the Kruskal-Wallis equality-of-populations rank test indicated the equality of distribution of all measured characteristics across all treatments. Therefore, demographic effects cannot explain differences in WTP between treatments.

### Results of Experiment 3

**Fig 3:**
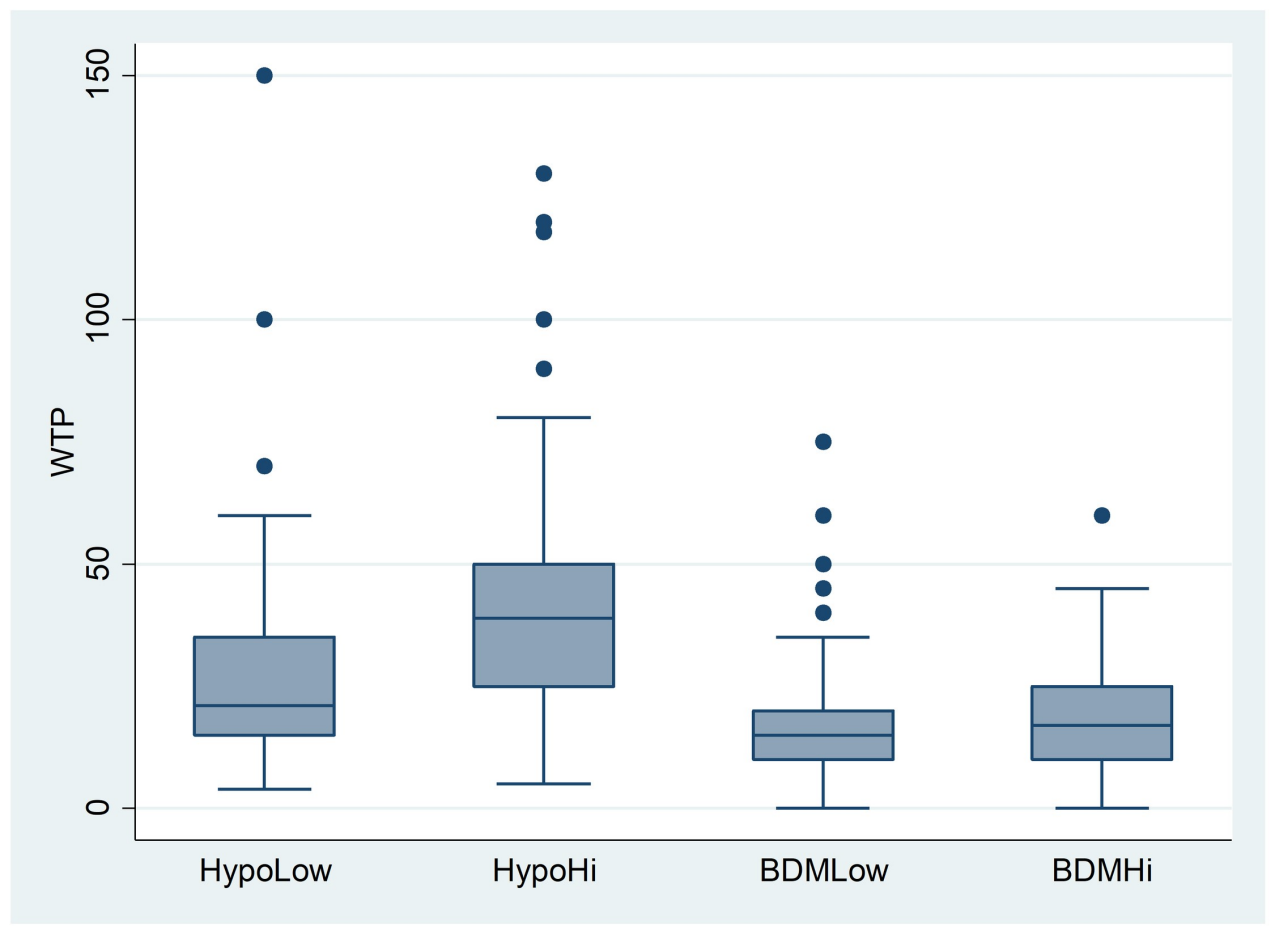
Experiment 3: WTPs by treatment (in PLN).

The mean value of WTP for a jar of flavored honey across the sample was equal to 26.01 PLN (5.74 EUR), with a median value of 20 PLN and a standard deviation of 19.53 PLN (for a histogram, see S5 Fig). One observation was removed because it was ambiguous ("500 to 1000 = > 2000”). As in Experiments 1 and 2, we started our analysis by comparing the WTP values in each treatment; see Table 12 and Fig 3.

**Table 12:** Experiment 3: WTPs by treatment (in PLN).

	HypoLow	HypoHi	RealLow	RealHi
Mean	28.00	41.41	15.76	17.96
Median	21	39	15	17
Std. Dev.	19.81	22.21	10.33	10.31
N	204	205	196	193

Similarly to before, WTP values in hypothetical treatments were higher than in BDM treatments and the anchor affected the valuation in the Hypo condition only. The differences between the WTP values in HypoLow vs. BDMLow and in HypoHi vs. BDMHi were highly significant (*z* = 8.944, *p*<.0001; *z* = 12.625, *p*<.0001), a clear HB. As for the anchoring effect, this time it was significant not only in the Hypo condition (*z* = −7.856, *p* = <0.0001) but also in the BDM condition (*z* = −2.837, *p* = 0.0046), although in the latter it was much weaker (48% vs. 14%).

As in previous experiments, to explicitly test for interactions between the effects under investigation, we estimated the OLS regression models with the same dependent variable as in Experiment 2 (WTP value). Variable labels are provided in Table 13.

**Table 13:** Experiment 3: Variable labels.

hypothetical_dummy	1—for declarative (hypothetical) valuation,
	0—for real valuation
high_anchor_dummy	1—for a high anchor,
	0—for a low anchor
hypothetical#high_anchor_interaction	interaction between hypothetical and high_anchor_dummies
male	1- male,
	0—female
Age	participant’s age
higher_education	1—if the participant has higher education,
	0—in all other cases
unemployed	1- if the participant is unemployed,
	0 –otherwise
gift	1—if the participant would like to use the honey as a gift,
	0—in all other cases
attractive	1- if the selected honey is attractive or very attractive for the participant,
	0—in all other cases
likes_honey_very_much	1- if the participant likes honey very much,
	0—in all other cases
experimenter	1—if the experiment was conducted by experimenter no. 1,
	0—if the experiment was conducted by experimenter no. 2
honey_ginger:	1- if participant selected the honey with garlic and ginger;
	0 –otherwise
honey_cocoa	1- if participant selected the honey with cocoa,
	0 –otherwise

Again, dependent variable was logarythmised to obtain the correct functional form of the models. We used White’ heteroskedasticity consistent estimators. Models 1–3 were very similar to those of Experiment 2. In model (4), we additionally included the extent to which the participants liked the presented honey and how much they liked honey in general, as well as whether they intended to offer the honey as a gift. Model (5), again, included the dummy for the experimenter who carried out the particular experiment and which flavour was selected by participants. Table 14 presents the results.

**Table 14:** Experiment 3: Regression table: WTP values.

	(1)	(2)	(3)	(4)	(5)
**hypothetical_dummy**	0.776***	0.632***	0.625***	0.562***	0.572***
**high_anchor_dummy**	0.287***	0.138	0.146	0.140	0.144
**hypothetical#high_anchor_interaction**		0.289***	0.289***	0.292***	0.289***
**male**			0.014	0.023	0.017
**age**			-0.002	-0.003	-0.003**
**higher_education**			-0.016	0.008	0.014
**unemployed**			-0.214***	-0.201***	-0.193***
**gift**				0.007	0.004
**attractive**				0.431***	0.426***
**likes_honey_very_much**				0.046	0.047
**experimenter**					0.033
**honey_ginger**					-0.012
**honey_cocoa**					-0.001
**cons**	793	793	786	786	784
	0.2280	0.2349	0.2457	0.2861	0.2890
**N**	110.56	89.28	43.73	35.10	29.05
**R-sqr**	0.0000	0.0000	0.0000	0.0000	0.0000
**F**	793	793	786	786	784
**Prob>F**	0.2280	0.2349	0.2457	0.2861	0.2890

** p < .05

*** p < .01

The interaction between the two variables of interest was highly significant whenever it was included. The other significant variables in our models influenced the valuation in similar ways as in Experiment 2. Again, our results were confirmed by Wald tests (F(1, 789) = 7.16; p = 0.0076), see S8 Appendix.

Again, the results were robust to the exclusion of inconsistencies. This time, they were slightly more numerous, representing 4.13% of all responses. The prevalence was once again higher in the hypothetical condition: (7.84% in HypoLow vs. 4.08% in BDMLow and 3.90% in HypoHi vs 0.52% in BDMHi); the latter difference was significant (*z* = 2.267, *p* = .0234).

The interaction effect was also confirmed in the permutation ANOVA, see Table 15.

**Table 15:** Experiment 3: Permutation ANOVA.

	SS	Df	F	Parametric P(>F)	Permutation P(>F)
hypothetical	14980.1	1	53.676	0.0000	0.0002
high_anchor	467.7	1	1.676	0.1958	0.1928
hypothetical*high_anchor	6268.1	1	22.460	0.0000	0.0002
Residuals	221592.3	794			

## Conclusions

The results of our experiments were remarkably consistent, although they involved three different products and two different settings: Experiment 1 was conducted in the lab and Experiments 2 and 3 in the field. Laboratory experiments, with their “sterile environment”, yield the greatest possible control and observability [58]. However, predominantly due to their artificiality and limited sample diversity, their findings may not translate well into non-lab settings, a rationale for various types of field experiments [59]. It is when both approaches are used and yield consistent results (as in our project) that we can be most certain about their internal and external validity.

We have strongly confirmed Hypothesis 1: HB influencing the valuation of a good. One reason for this tendency is that practical considerations may receive insufficient scrutiny when dealing with hypothetical choices. In our case, participants in the Hypo condition (of Experiment 1) were significantly less likely to state that they considered whether they actually needed the good than those in the BDM condition (instead, they reported having devoted relatively more attention to the technique, the description of the artist, and examples of her works). Social desirability bias, intention-behaviour discrepancy and experimenter demand effects may also have played some role. As a result, the participant may have overstated their actual WTP in the hypothetical condition. Hypothesis 2, predicting that anchoring influences the valuation of a good, was partially confirmed, as the effect was only found in the hypothetical condition (except for a weak effect in Experiment 3); consequently, Hypothesis 3 (the interaction) was confirmed. This finding appears to be in opposition to the strand of research implying that the standard anchoring mechanism has little to do with insufficient mental effort [48]. This theory would lead to the incorrect prediction of the weak effect of providing financial incentives to report truthfully. It also contradicts the aforementioned inadequately documented evidence, supposedly demonstrating that anchors continue to work when responses are real, which can be found in previous literature.

To the extent that susceptibility to anchoring is a bad sign concerning data quality, our findings speak against the validity of hypothetical methods. This is corroborated by our additional observation of the greater prevalence of inconsistent responses in the hypothetical condition.

Indeed, if anchors have much more impact on hypothetical than real decisions, providing a low one could be a promising tool in curbing HB, which could selectively reduce the reported valuations of those responders who tend to overstate them compared to their real WTP. To illustrate how this could work, we sketch a very simple model. Every participant knows his/her true WTP. Some of them always report it truthfully. The others are ashamed that their WTP is so low. They only report it truthfully if they have (sufficiently strong) incentives to do so or if they receive a signal suggesting that there is no need to be ashamed. An anchor lower than or equal to their WTP sends just such a signal. This model explains both that valuations tend to be higher and more affected by anchors in the hypothetical setting. It also predicts that a low anchor (lower or equal than the lowest WTP in the sample) improves the quality of hypothetical data, making it identical to real data. Of course, this is just a toy model, which may be far from reality in many contexts.

In our experiments, we only investigated private goods. Given that HB seems to be more pronounced in public goods [10, 12], the quality of data being lower due to its hypothetical nature may be a particular problem there. Moreover, incentivizing preference for public goods is very often more difficult than in the case of private goods and then, hypothetical methods are only ones that allow to estimate the economic value of goods and services. Our findings thus emphasize the importance of developing and implementing alternative ways of making declared preference for public goods consequential, such as the choice-matching approach of Cvitanić et al. [60]. WTP for public goods in contingent valuation tasks may be also affected by anchoring [34, 61, 62]. However, we are not aware of any direct comparison of the size of the anchoring effect in experiments with private vs. public goods; therefore we can only suspect that the effect of interaction between hypothetical bias and anchoring effect may also be present in public goods context. Of course, to verify our conjecture another study with public goods or services should be conducted.

## Supporting information

S1 Appendix Experiment 1: Transcript of instructions. (DOCX)

S2 Appendix Experiment 1: Transcript of the questionnaire. (DOCX)

S3 Appendix Experiment 1: Wald test. (DOCX)

S4 Appendix Experiment 1: OLS models in sub-samples. (DOCX)

S5 Appendix Experiments 2 and 3: Transcript of instructions. (DOCX)

S6 Appendix Experiments 2 and 3: Transcript of a questionnaire. (DOCX)

S7 Appendix Experiment 2: Wald test. (DOCX)

S8 Appendix Experiment 3: Wald test. (DOCX)

S1 Fig Experiment 1: Histogram of WTP values across the sample. (PDF)

S2 Fig Experiment 2: Selected products. (JPG)

S3 Fig Experiment 2: Histogram of WTP values across the sample. (PDF)

S4 Fig Experiment 3: Selected products. (JPG)

S5 Fig Experiment 3: Histogram of the WTP values across the sample. (PDF)
