# Supplementary material for: Anchors on prices of consumer goods only hold when decisions are hypothetical
Source: PLoS One. 2022 Jan 5;17(1):e0262130. doi: 10.1371/journal.pone.0262130 (PMC8730394; doi:10.1371/journal.pone.0262130)
Supplement: S1 Appendix — (DOCX) [file pone.0262130.s001.docx]

**S1 Appendix. Experiment 1: transcript of instructions**

Part 1: Introduction [all treatments, presented orally]

Welcome!

Thank you for taking part in our experiment! One randomly selected participant will receive a 50 PLN Rossmann voucher today.

Please turn off your mobile phones, remain silent, and do not communicate with one another. Conducting conversations, looking around the room, and showing your answers to others is not allowed.

If you have any questions during the experiment, raise your hand and wait for the experimenter to come to you. (Do not ask your question aloud!)

People who do not comply with these rules will be excluded from the experiment and lose the chance of winning the prize.

We would like to inform you that the study is anonymous and all data collected will be used solely for scientific purposes. Along with the next set of instructions, you will receive an individual respondent code that you will need until the end of the experiment. Place it in a prominent spot on the bench/desktop in front of you and keep it until the end of the experiment.

Part 2: Rules [HypoLow, HypoHi]

In a moment, you will receive a questionnaire in which you will be asked to value a product. In the questionnaire, you will have to specify the maximum price you would be willing to pay for this product. Your reply will be purely declarative and no real transactions will be made on this basis. The amount you specify will not be disclosed to anyone.

If you have any questions, raise your hand and wait for the experimenter to approach you.

Part 2: Rules: [RealLow, RealHi]

**In a moment, you will have the opportunity to buy a product.** You will receive a valuation questionnaire in which you will be asked to specify the maximum price you would be willing to pay for this product. The amount you give will not be disclosed to anyone.

**Your reply will be binding and may determine whether you are able to buy the product.** After everyone gives their amounts, three participants will be selected. If you are one of them, a transaction price will be drawn for you. If it is lower than or equal to the amount you specified, **you will be required to buy the product at the drawn price;** however, if the drawn transaction price is higher than the amount you provided, no transaction will take place.

**The best thing you can do in this situation is to give your actual valuation, which is the maximum price you are willing to pay for the presented product**. If you give an amount higher than your actual valuation, you may have to pay more than you are willing to. If, on the other hand, you give a lower valuation than your actual one, you may be disappointed with your inability to purchase the product at your actual price.

Example: The participant declares that the maximum price s/he is willing to pay for the product is 15 000 PLN; a price of 12 000 PLN is drawn. The participant buys the product for 12 000 PLN; however, if s/he declared only 11 000 PLN, s/he would not be able to buy the product. Of course, this is just an example; the amounts involved in the experiment will be significantly lower.

If you are required to make a purchase and you do not have enough money with you, you will be able to pay up to a week after the experiment; thus even if you do not have cash on you, you can still participate in the experiment.

If you have any questions, raise your hand and wait for the experimenter to approach you.

Part 3: Information about the product and valuation questionnaire [HypoLow, HypoHi]

Voucher for the professional caricature or portrait

The portrait or caricature will be made by a professional artist – Agnieszka Paczuska, a Warsaw portraitist and painter, with an arts education and 20 years’ experience.

- a caricature or portrait showing you or a person of your choice (e.g., as a gift)

- A4 format

- technique: pencil/coal/mixed technique (black-and-white)

- drawn based on the photo you selected or in person in the artist’s studio (in Warsaw) at a convenient time

- voucher to be valid for 3 months from the date of receipt

**…………………………………………………………………………………………………..**

Answer the following questions:

Would you, hypothetically, buy the presented voucher for 20 PLN [80 PLN] ?

- YES
- NO

Give the maximum price that, hypothetically, you would be willing to pay for the presented voucher. (Please enter a specific amount in PLN.)

Part 3: Information about the product and valuation questionnaire [RealLow, RealHi]

Voucher for the professional caricature or portrait

The portrait or caricature will be made by a professional artist – Agnieszka Paczuska, a Warsaw portraitist and painter, with an arts education and 20 years’ experience.

- a caricature or portrait showing you or a person of your choice (e.g., as a gift)

- A4 format

- technique: pencil/coal/mixed technique (black-and-white)

- drawn based on a photo you have selected or in person in the artist’s studio (in Warsaw) at a convenient time

- voucher to be valid for 3 months from the date of receipt

**…………………………………………………………………………………………………..**

**Remembering that your answers are binding (and will depend on whether you are able to buy the product or not),** answer the following questions:

Would you buy the presented voucher for 20 PLN [80 PLN] ?

- YES
- NO

Give the maximum price that you would be willing to pay for the presented voucher. (Please enter a specific amount in PLN.)
