# Supplementary material for: Anchors on prices of consumer goods only hold when decisions are hypothetical
Source: PLoS One. 2022 Jan 5;17(1):e0262130. doi: 10.1371/journal.pone.0262130 (PMC8730394; doi:10.1371/journal.pone.0262130)
Supplement: S2 Appendix — (DOCX) [file pone.0262130.s002.docx]

**S2 Appendix. Experiment 1: transcript of the questionnaire**

1. Have you ever bought a caricature or a portrait before?

- YES
- NO

2. What factor(s) did you take into consideration when evaluating the voucher during the experiment? (You can choose up to 3 relevant answers.)

- Work technique
- Work format
- Time to use the voucher
- Examples of the artist's works
- Attached information about the artist
- Whether you need this type of product
- How much you can afford to pay for it
- Other (please specify)……………………………..

3. Did you like the example works of the artist?

- Definitely yes
- Yes
- Neither yes nor no
- No
- Definitely not

4. How would you use the presented voucher?

- For myself
- As a gift
- I don’t know

5. Are you interested in art?

- Definitely yes
- Yes
- Neither yes nor no
- No
- Definitely no

6.What do you think is the average market price of a black and white caricature in A4 format?

(enter a specific amount in PLN)

….....................…………………………

7. What do you think is the average market price of a black and white portrait in A4 format?

(enter a specific amount in PLN)

…......................................................

8. Gender

- Female
- Male

9. Year of birth

…………………………

10. Are you in a relationship?

- Yes, I have a boyfriend/girlfriend
- Yes, I have a fiancé/fiancée
- Yes, I have a husband/wife
- No, I am single

11. Do you work currently?

- Yes
- No

12. How would you evaluate your financial situation?

- I don’t have enough money for the most urgent needs
- I have to deny myself many things to keep enough money for living
- I have enough money for everyday living, but I can’t afford higher expenses
- I have enough money for all expenses, and I can save
- I am wealthy, I do not have to save even for larger expenses
- I don’t know/it's hard to say

13. Where were you born?

- a village
- a city with less than 100,000 residents
- a city with over 100,000 residents
