# Supplementary material for: Anchors on prices of consumer goods only hold when decisions are hypothetical
Source: PLoS One. 2022 Jan 5;17(1):e0262130. doi: 10.1371/journal.pone.0262130 (PMC8730394; doi:10.1371/journal.pone.0262130)
Supplement: S3 Appendix — (DOCX) [file pone.0262130.s003.docx]

**S3 Appendix. Experiment 1: Wald test**

**Source |       SS           df       MS      Number of obs   =       211**

**-------------+----------------------------------   F(4, 207)       =    765.16**

**Model |  2754.17535         4  688.543837   Prob > F        =    0.0000**

**Residual |  186.273305       207  .899871039   R-squared       =    0.9367**

**-------------+----------------------------------   Adj R-squared   =    0.9354**

**Total |  2940.44865       211  13.9357756   Root MSE        =    .94862**

**------------------------------------------------------------------------------**

**lWTP |      Coef.   Std. Err.      t    P>|t|     [95% Conf. Interval]**

**-------------+----------------------------------------------------------------**

**_IHypoLow_1 |    3.96998   .1328327    29.89   0.000     3.708101    4.231858**

**_IHypoHi_1 |    4.39696   .1315493    33.42   0.000     4.137612    4.656308**

**_IBDMLow_1 |   2.820902   .1267641    22.25   0.000     2.570988    3.070816**

**_IBDMHi_1 |   3.099097   .1315493    23.56   0.000     2.839749    3.358445**

**------------------------------------------------------------------------------**

**. test _IHypoLow_1 + _IBDMHi_1 = _IHypoHi_1 + _IBDMLow_1**

**( 1)  _IHypoLow_1 - _IHypoHi_1 - _IBDMLow_1 + _IBDMHi_1 = 0**

**F(  1,   207) =    0.32**

**Prob > F =    0.5698**
