# Supplementary material for: Anchors on prices of consumer goods only hold when decisions are hypothetical
Source: PLoS One. 2022 Jan 5;17(1):e0262130. doi: 10.1371/journal.pone.0262130 (PMC8730394; doi:10.1371/journal.pone.0262130)
Supplement: S4 Appendix — (DOCX) [file pone.0262130.s004.docx]

**S4 Appendix. Experiment 1: OLS models in sub-samples**

1. **OLS model in sub-sample with BDM**

|  | **(1)** | **(2)** | **(3)** | **(4)** |
| --- | --- | --- | --- | --- |
| **high_anchor_dummy** | 0.278 | 0.281 | 0.328 | 0.379 |
| **male** |  | 0.308 | 0.370 | 0.317 |
| **age** |  | -0.115 | -0.096 | -0.111 |
| **in_relationship** |  | 0.248 | 0.222 | 0.161 |
| **unemployed** |  | -0.173 | -0.151 | -0.210 |
| **city** |  | -0.092 | -0.045 | -0.051 |
| **financial_situation** |  | 0.282 | 0.254 | 0.336 |
| **likes_examples** |  |  | 0.387 | 0.368 |
| **gift** |  |  | 0.074 | 0.043 |
| **art** |  |  | 0.035 | 0.011 |
| **price_caricature** |  |  |  | 0.003 |
| **price_portrait** |  |  |  | -0.003 |
| **cons** | 2.821*** | 4.860** | 4.147** | 4.559** |
|  |  |  |  |  |
| **N** | 108 | 108 | 108 | 103 |
| **R-sqr** | 0.0176 | 0.0885 | 0.1204 | 0.1549 |
| **F** | 1.89 | 1.39 | 1.33 | 1.69 |
| **Prob>F** | 0.1717 | 0.2193 | 0.2269 | 0.0813 |

** p<.05, *** p<.01

1. **OLS models in sub-sample with hypothetical valuation**

|  | **(1)** | **(2)** | **(3)** | **(4)** |
| --- | --- | --- | --- | --- |
| **high_anchor_dummy** | 0.427** | 0.441*** | 0.471*** | 0.431*** |
| **male** |  | -0.107 | -0.049 | 0.027 |
| **age** |  | -0.075 | -0.055 | -0.032 |
| **in_relationship** |  | 0.251 | 0.185 | 0.119 |
| **unemployed** |  | 0.463** | 0.466** | 0.372** |
| **city** |  | -0.203 | -0.152 | -0.230 |
| **financial_situation** |  | 0.066 | 0.029 | 0.076 |
| **likes_examples** |  |  | 0.474*** | 0.409** |
| **gift** |  |  | -0.087 | -0.037 |
| **art** |  |  | 0.281 | 0.183 |
| **price_caricature** |  |  |  | 0.001 |
| **price_portrait** |  |  |  | 0.003** |
| **cons** | 3.970*** | 5.153*** | 4.335** | 3.605** |
|  |  |  |  |  |
| **N** | 103 | 103 | 103 | 102 |
| **R-sqr** | 0.0633 | 0.1621 | 0.2812 | 0.4529 |
| **F** | 6.82 | 2.63 | 3.60 | 7.34 |
| **Prob>F** | 0.0104 | 0.0160 | 0.0005 | 0.0000 |
