# Supplementary material for: Anchors on prices of consumer goods only hold when decisions are hypothetical
Source: PLoS One. 2022 Jan 5;17(1):e0262130. doi: 10.1371/journal.pone.0262130 (PMC8730394; doi:10.1371/journal.pone.0262130)
Supplement: S5 Appendix — (DOCX) [file pone.0262130.s005.docx]

**S5 Appendix. Experiments 2 and 3: transcript of instructions**

Part 1: Introduction [all treatments, presented orally]

Welcome!

We are researchers from the University of Warsaw. We are conducting a survey, which will only take a few minutes. I am wondering if you would like to participate. At the end of this survey, you will play a game in which you can win a Westfield Arkadia Gift Card, worth 100 PLN. If you have any questions during the survey, do not hesitate to ask.

We would like to inform you that the study is anonymous and all data collected will be used solely for scientific purposes.

Part 2: Rules [HypoLow, HypoHi]

In a moment, you will receive a questionnaire in which you will be asked to value the presented product. In the questionnaire, you will have to specify the maximum price you would be willing to pay for this product. It will be purely declarative and no real transactions will be made on this basis.

Part 2: Rules: [RealLow, RealHi]

**In a moment, you will have the opportunity to buy the presented product.** You will receive a valuation questionnaire in which you will be asked to specify the maximum price you would be willing to pay for this product.

**Your choice will be binding: it will determine whether you buy the product.** After you state your amount, a transaction price will be drawn for you. If it is lower than or equal to the amount you specified, **you will be required to buy the product at the drawn price;** however, if the drawn transaction price is higher than the amount you provided, no transaction will take place.

**The best thing you can do in this situation is to give your actual valuation, which is the maximum price you are willing to pay for the presented product**. If you give an amount higher than your actual valuation, you may have to pay more than you are willing to. If, on the other hand, you give a lower valuation than your actual one, you may be disappointed with your inability to purchase the product offered at an acceptable price.

Example: The participant declares that the maximum price s/he is willing to pay for the product is 15 000 PLN; a price of 10 000 PLN is drawn. The participant buys the product for 10 000 PLN; however, if the price was 20 000 PLN, s/he would not be able to buy the product. Of course, this is just an example; the amounts involved in the experiment will be significantly lower.

Part 3: Information about the product and valuation questionnaire [HypoLow, HypoHi]

[Short information about the product]

Answer the following questions:

Would you, hypothetically, buy the selected product for 10 PLN [60 PLN]?

- YES
- NO

Give the maximum price that, hypothetically, you would be willing to pay for the presented product. (Please enter a specific amount in PLN.)

Part 3: Information about the product and valuation questionnaire [RealLow, RealHi]

[Short information about product]

**Remembering that your answers are binding and whether you buy the product will depend on them,** answer the following questions:

Would you buy the presented product for 10 PLN [60 PLN]?

- YES
- NO

Give the maximum price that you are willing to pay for the presented product. (Please enter a specific amount in PLN.)
