# Supplementary material for: Anchors on prices of consumer goods only hold when decisions are hypothetical
Source: PLoS One. 2022 Jan 5;17(1):e0262130. doi: 10.1371/journal.pone.0262130 (PMC8730394; doi:10.1371/journal.pone.0262130)
Supplement: S6 Appendix — (DOCX) [file pone.0262130.s006.docx]

**S6 Appendix. Experiments 2 and 3: transcript of a questionnaire**

1. How would you use the presented product?

- For myself
- As a gift
- I don’t know

2. Do you like this product?

- Definitely yes
- Yes
- Neither yes nor no
- No
- Definitely not

3. Gender

- Female
- Male

4. Year of birth

…………………………

5. Education level:

- Primary
- Vocational
- Secondary
- Higher

6. Do you work currently?

- Yes
- No
