# Supplementary material for: Anchors on prices of consumer goods only hold when decisions are hypothetical
Source: PLoS One. 2022 Jan 5;17(1):e0262130. doi: 10.1371/journal.pone.0262130 (PMC8730394; doi:10.1371/journal.pone.0262130)
Supplement: S7 Appendix — (DOCX) [file pone.0262130.s007.docx]

**S7 Appendix. Experiment 2: Wald test**

**Source |       SS           df       MS      Number of obs   =       775**

**-------------+----------------------------------   F(4, 771)       =   2580.37**

**Model |  7117.36329         4  1779.34082   Prob > F        =    0.0000**

**Residual |  531.658024       771  .689569421   R-squared       =    0.9305**

**-------------+----------------------------------   Adj R-squared   =    0.9301**

**Total |  7649.02131       775  9.86970492   Root MSE        =     .8304**

**------------------------------------------------------------------------------**

**lWTP |      Coef.   Std. Err.      t    P>|t|     [95% Conf. Interval]**

**-------------+----------------------------------------------------------------**

**_IHypoLow_1 |   3.282234   .0588657    55.76   0.000     3.166678     3.39779**

**_IHypoHi_1 |   3.711135   .0591638    62.73   0.000     3.594994    3.827276**

**_IBDMLow_1 |   2.447698   .0597737    40.95   0.000      2.33036    2.565037**

**_IBDMHi_1 |   2.436318   .0608881    40.01   0.000     2.316792    2.555844**

**. test _IHypoLow_1 + _IBDMHi_1 = _IHypoHi_1 + _IBDMLow_1**

**( 1)  _IHypoLow_1 - _IHypoHi_1 - _IBDMLow_1 + _IBDMHi_1 = 0**

**F(  1,   771) =   13.61**

**Prob > F =    0.0002**
