# Supplementary material for: Anchors on prices of consumer goods only hold when decisions are hypothetical
Source: PLoS One. 2022 Jan 5;17(1):e0262130. doi: 10.1371/journal.pone.0262130 (PMC8730394; doi:10.1371/journal.pone.0262130)
Supplement: S8 Appendix — (DOCX) [file pone.0262130.s008.docx]

**S8 Appendix. Experiment 3: Wald test**

**Source | SS df MS Number of obs = 793**

**-------------+---------------------------------- F(4, 789) = 3142.64**

**Model | 7269.3327 4 1817.33317 Prob > F = 0.0000**

**Residual | 456.264523 789 .578282032 R-squared = 0.9409**

**-------------+---------------------------------- Adj R-squared = 0.9406**

**Total | 7725.59722 793 9.74224114 Root MSE = .76045**

**------------------------------------------------------------------------------**

**lWTP | Coef. Std. Err. t P>|t| [95% Conf. Interval]**

**-------------+----------------------------------------------------------------**

**_IHypoLow_1 | 3.1606 .053242 59.36 0.000 3.056087 3.265113**

**_IHypoHi_1 | 3.58773 .053112 67.55 0.000 3.483473 3.691988**

**_IBDMLow_1 | 2.528939 .0547383 46.20 0.000 2.421489 2.636388**

**_IBDMHi_1 | 2.666879 .0550241 48.47 0.000 2.558868 2.77489**

**------------------------------------------------------------------------------**

**. test _IHypoLow_1 + _IBDMHi_1 = _IHypoHi_1 + _IBDMLow_1**

**( 1) _IHypoLow_1 - _IHypoHi_1 - _IBDMLow_1 + _IBDMHi_1 = 0**

**F( 1, 789) = 7.16**

**Prob > F = 0.0076**
