# Supplementary figures and images for: Anchors on prices of consumer goods only hold when decisions are hypothetical
Source: PLoS One. 2022 Jan 5;17(1):e0262130. doi: 10.1371/journal.pone.0262130 (PMC8730394; doi:10.1371/journal.pone.0262130)

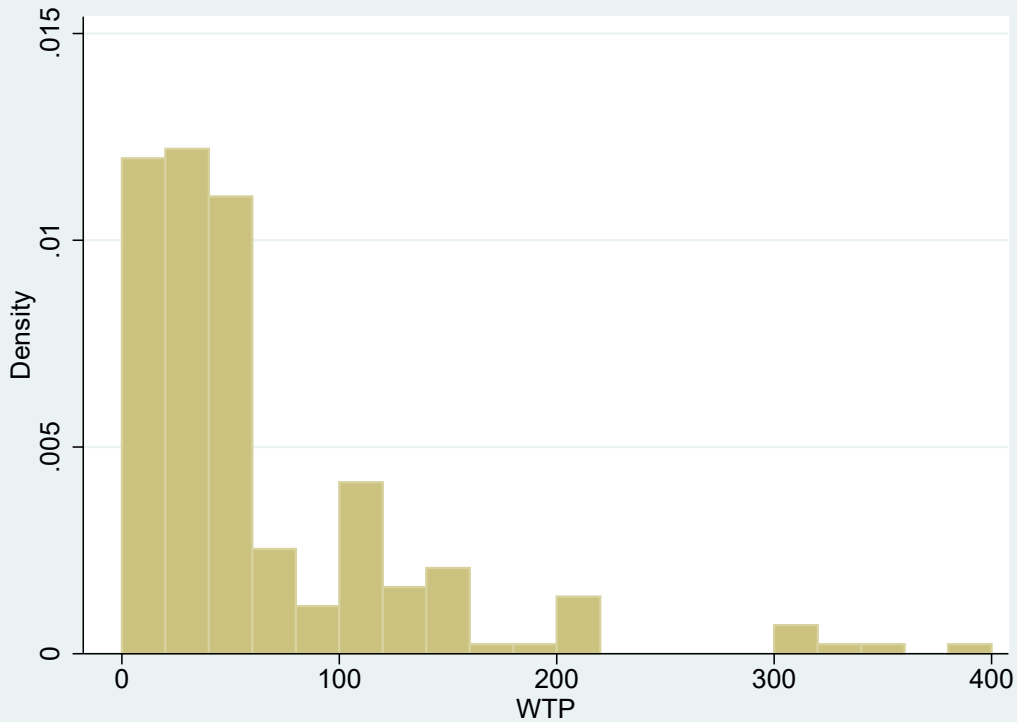

Supplement: S1 Fig — (PDF) [file pone.0262130.s009.pdf]

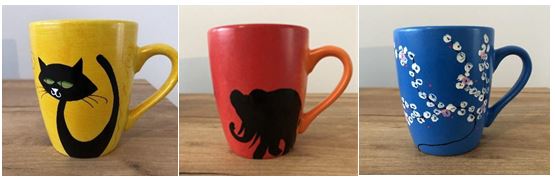

Supplement: S2 Fig — (JPG) [file pone.0262130.s010.JPG]

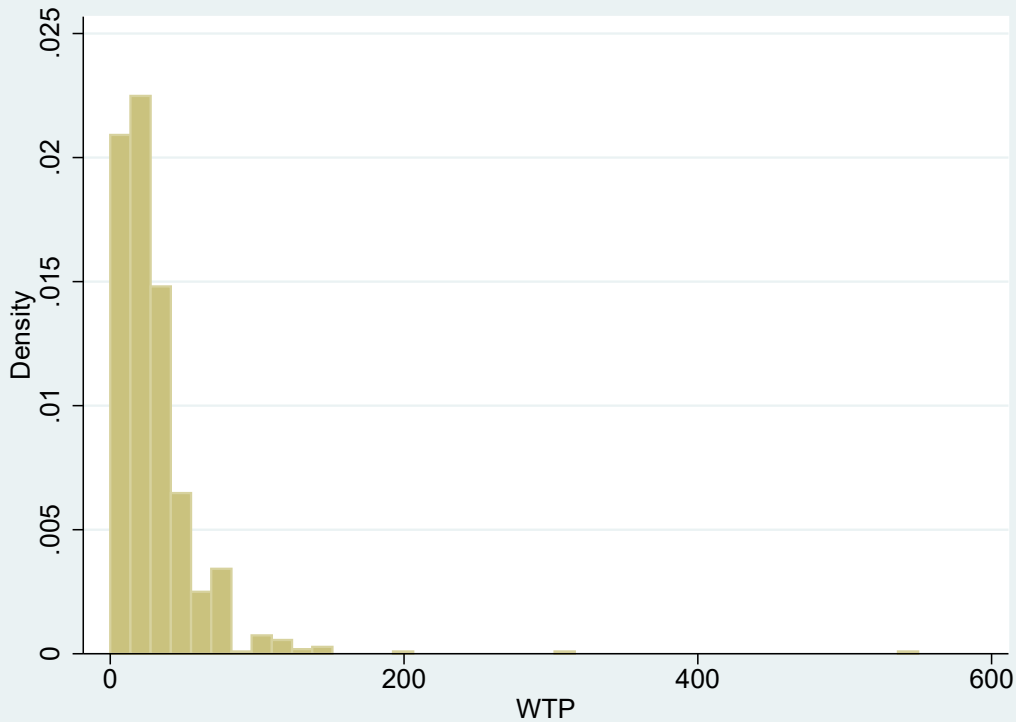

Supplement: S3 Fig — (PDF) [file pone.0262130.s011.pdf]

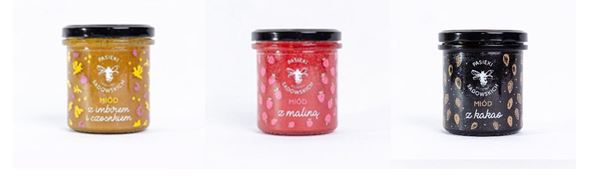

Supplement: S4 Fig — (JPG) [file pone.0262130.s012.JPG]

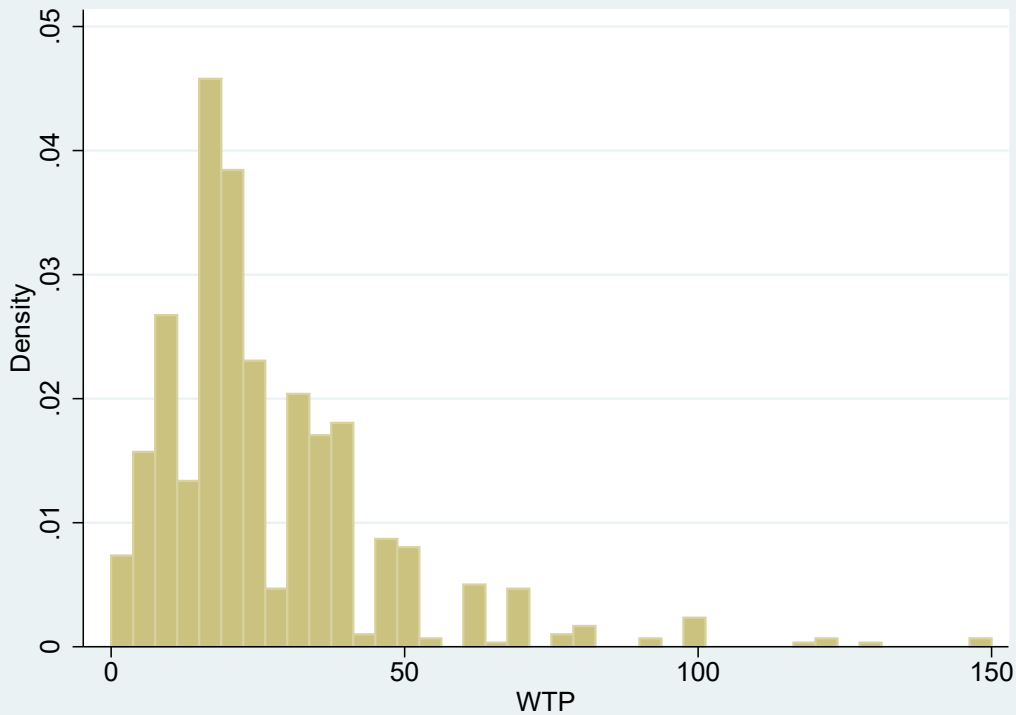

Supplement: S5 Fig — (PDF) [file pone.0262130.s013.pdf]
